# Flipping the script: Understanding riboswitches from an alternative perspective

**DOI:** 10.1016/j.jbc.2024.105730

**Published:** 2024-02-08

**Authors:** Lukasz T. Olenginski, Savannah F. Spradlin, Robert T. Batey

**Affiliations:** Department of Biochemistry, University of Colorado, Boulder, Colorado, USA

**Keywords:** riboswitch, gene regulation, strand exchange, RNA folding, RNA structure

## Abstract

Riboswitches are broadly distributed regulatory elements most frequently found in the 5′-leader sequence of bacterial mRNAs that regulate gene expression in response to the binding of a small molecule effector. The occupancy status of the ligand-binding aptamer domain manipulates downstream information in the message that instructs the expression machinery. Currently, there are over 55 validated riboswitch classes, where each class is defined based on the identity of the ligand it binds and/or sequence and structure conservation patterns within the aptamer domain. This classification reflects an “aptamer-centric” perspective that dominates our understanding of riboswitches. In this review, we propose a conceptual framework that groups riboswitches based on the mechanism by which RNA manipulates information directly instructing the expression machinery. This scheme does not replace the established aptamer domain-based classification of riboswitches but rather serves to facilitate hypothesis-driven investigation of riboswitch regulatory mechanisms. Based on current bioinformatic, structural, and biochemical studies of a broad spectrum of riboswitches, we propose three major mechanistic groups: (1) “direct occlusion”, (2) “interdomain docking”, and (3) “strand exchange”. We discuss the defining features of each group, present representative examples of riboswitches from each group, and illustrate how these RNAs couple small molecule binding to gene regulation. While mechanistic studies of the occlusion and docking groups have yielded compelling models for how these riboswitches function, much less is known about strand exchange processes. To conclude, we outline the limitations of our mechanism-based conceptual framework and discuss how critical information within riboswitch expression platforms can inform gene regulation.

Riboswitches are a ubiquitous means of genetic regulation in bacteria defined by the ability of an RNA element to directly bind a small molecule or ion to control RNA synthesis, expression, or metabolism (1, 2). This regulatory activity requires two distinct components: a ligand-binding structure called the aptamer domain and a downstream sequence referred to as the expression platform that directs the regulatory response based on the occupancy status of the aptamer domain (*i.e.*, ligand bound or unbound) (Fig. 1) (3). Since their initial discovery (4, 5, 6), riboswitches have been classified by two properties of their aptamer domain: the identity of the ligand it binds and/or conservation patterns of its primary and secondary structure (7, 8). With this categorization scheme, over 55 distinct riboswitch classes have been described, and this number continues to increase, albeit at a slower pace as de-orphaning RNA elements that appear to have the characteristics of riboswitches becomes more difficult (9, 10).

**Figure 1:**
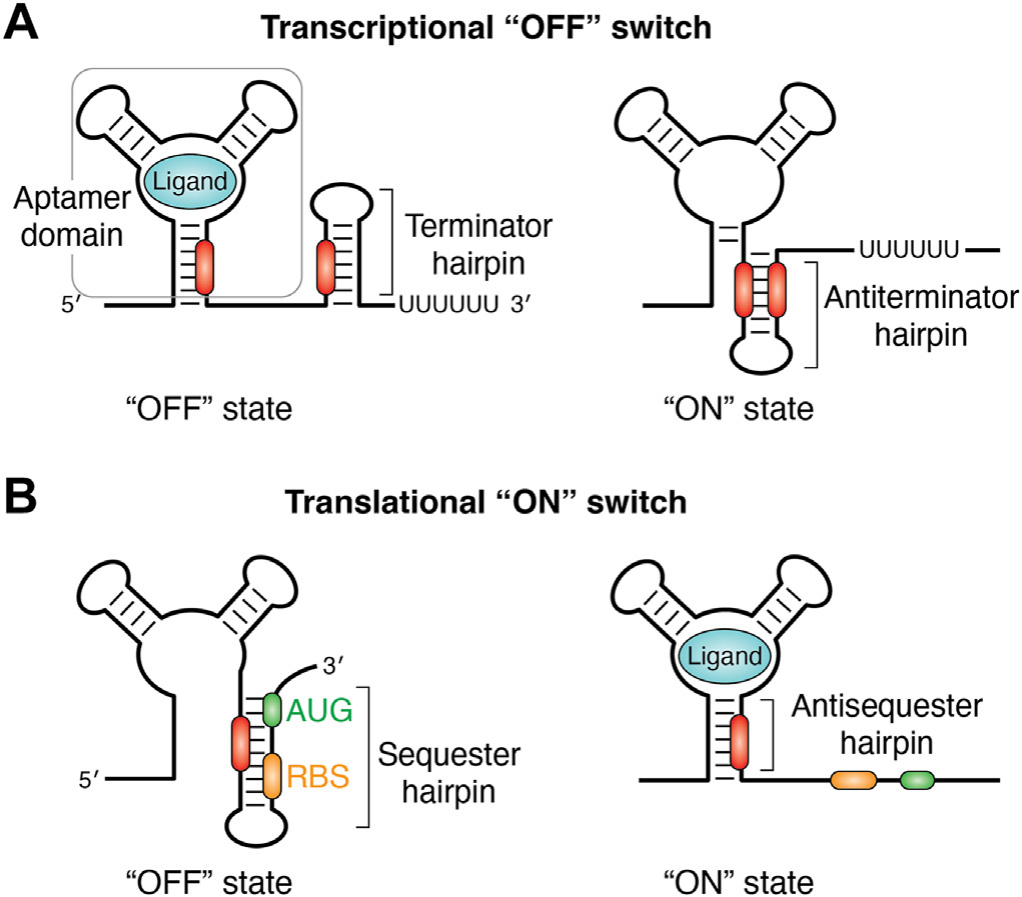
**Schematic of riboswitch genetic regulatory mechanisms.***A*, schematic of the structural switch of a riboswitch under transcriptional control. In the bound state (*left*), ligand binding to the aptamer domain (*gray box*) causes formation of a terminator hairpin, preventing transcription. In the absence of ligand binding (*right*), the complementary RNA regions (*red*) form an antiterminator hairpin, resulting in active transcription. This cartoon is modeled from the *Bacillus subtilis xpt-pbuX* guanine-responsive purine riboswitch (189). *B*, schematic of the structural switch of a riboswitch under translational control. In the absence of ligand binding (*left*), the RBS (*orange*) and AUG start codon (*green*) are occluded within a sequester hairpin, preventing translation. In the bound state (*right*), ligand binding to the aptamer domain induces the formation of an antisequester hairpin, enabling the ribosome access to the RBS and AUG to initiate translation. This cartoon is modeled from the *Vibrio vulnificus add* adenine-responsive purine riboswitch (190). RBS, ribosome-binding site.

However, this classification reflects an “aptamer-centric” perspective that forces our understanding of riboswitches to be viewed through the lens of their aptamer domains rather than their expression platforms. This emphasis is driven by the fact that almost all riboswitch sequence and structural conservation patterns are found within the aptamer domain, enabling their discovery and classification using bioinformatic approaches (11). A second contributing factor to the aptamer-centric focus is that most mechanistic studies are derived from isolated riboswitch aptamer domains (*i.e.*, without the expression platform) (12, 13). Aptamer domain RNAs typically adopt highly stable structures that fold rapidly and with high fidelity (14), making them easy to study compared to other RNAs. In particular, these features have enabled facile determination of numerous atomic-level structures of aptamer domain-ligand complexes–most of which were elucidated shortly after their initial discovery (15, 16, 17, 18, 19, 20, 21). Currently, almost all riboswitch classes have at least one crystal or NMR structure of an aptamer domain-small molecule complex and associated biochemical data that assesses ligand-binding affinity and specificity (8). These structures have facilitated a diverse set of biophysical, biochemical, and computational studies that showcase the power of using riboswitch aptamer domains as model systems to explore RNA structure, folding, and small molecule recognition (22).

However, given that most of these studies have used truncated riboswitches that do not contain the expression platform, they often do not yield direct mechanistic insights into the coupling of ligand binding and regulatory activity. To direct the expression fate of a mRNA, a riboswitch must contain information within the expression platform that is presented to the expression machinery in a ligand-dependent fashion (3, 23). Typically, this is portrayed as a simple binary switch between two mutually exclusive secondary structures (Fig. 1). In this basic model, a sequence element from the aptamer domain can be incorporated into an alternative secondary structure within the expression platform, such as an antiterminator hairpin (Fig. 1*A*) or ribosome-binding site (RBS, alternatively known as the Shine Dalgarno sequence) sequester helix (Fig. 1*B*). Formation of these alternative secondary structures is dependent on the occupancy status of the aptamer domain. The ability of the expression platform to efficiently determine the occupancy status of the aptamer domain and establish an alternative conformation at the expense of the aptamer domain lies at the heart of the riboswitch’s ability to act as a regulator of mRNA expression. However, the mechanism(s) by which aptamer domains convey information to the expression platform is poorly understood compared to the much deeper knowledge of aptamer domain-small molecule recognition (24, 25). Understanding this binding-regulation linkage is the most important problem in conceptualizing the biology of riboswitches and their use in real-world applications.

Unlike the easily characterizable aptamer domains, analysis of riboswitch expression platforms has been significantly more difficult. One complicating factor is that expression platforms are highly variable in their sequence and structures, even among riboswitches that share a conserved aptamer domain. While bioinformatic analysis of expression platforms reveals specific features that are directly associated with the transcriptional (Fig. 1*A*) or translational (Fig. 1*B*) machinery, other functional elements that may be required for regulatory activity are obscure such as recruitment of protein factors or pausing of RNA polymerase (RNAP). Consequently, expression platforms are generally viewed through the lens of the expression machinery that they regulate (1, 26, 27), rather than the mechanism by which they present information to the expression machinery. A further complication is that many riboswitches only function in the context of transcription (27). Given that computational, biophysical, and biochemical approaches to investigate RNA folding and function in the context of transcription are currently limited (28, 29, 30), this aspect of riboswitch biology remains poorly understood. Despite these obstacles, there has been significant progress towards developing a mechanistic understanding of the linkage between the small molecule binding activity of the aptamer domain and the regulatory function of the expression platform.

In this review, we will discuss various emerging models of riboswitch function. To facilitate this discussion, we present riboswitches in a conceptual framework centered on how the occupancy status of the aptamer domain is mechanistically communicated to the expression platform. This perspective is neutral with regards to the nature of the ligand (and thus, the aptamer domain itself), the expression machinery that the riboswitch informs, and other protein components acting on the RNA. Put another way, we focus on the RNA itself rather than the molecules and factors acting on it. As a foundation for this perspective, we propose three primary groups of riboswitches based on how they mechanistically couple ligand binding to gene regulation: (1) “direct occlusion,” (2) “interdomain docking,” and (3) “strand exchange”. Each of these groups encompass multiple traditional aptamer-based riboswitch classes and should be seen as a means of contextualizing riboswitches complementary to the established classification scheme. For each group, we highlight the defining features that serve to inform how RNA sequence and structure transduces an input signal (*i.e.*, small molecule binding) into an output response (*i.e.*, level of gene expression).

### Group 1: Direct occlusion

Direct occlusion occurs when ligand binding to the aptamer domain directly blocks the expression machinery from accessing the message. This mechanism requires that the aptamer domain and expression platform be fully integrated into a single functional unit. In principle, this type of regulation could occur at the level of transcription *via* a Rho-independent (intrinsic) transcriptional terminator directly stabilized by a ligand. However, since the formation of the terminator hairpin occurs within the exit channel of RNAP to destabilize the elongation complex (31, 32), the timeframe for productive ligand binding would be extremely short–even if the ligand has easy access to the interior of RNAP to bind the hypothetical riboswitch. While these considerations make the prospect of finding such a riboswitch unlikely, the recently discovered guanidine-IV riboswitch may employ this mechanism. Guanidine binding appears to directly destabilize the intrinsic terminator by promoting the formation of a pseudoknot that involves the terminator’s terminal loop (33, 34).

At the translational level, expression can be regulated *via* ligand-dependent blocking of the RBS. In this model, the RBS is embedded within or immediately adjacent to the ligand-binding site such that the 30*S* ribosomal subunit is directly blocked from base pairing with the mRNA during translational initiation (Fig. 2*A*). This strategy is observed in a variety of riboswitches, including the SAM-II/V (35, 36), SAM-III (or SAM(MK)) (37), SAM-SAH (38), and selective members of the preQ_1_-I (pre-queuosine) (39, 40) and preQ_1_-II classes (41). In most cases, the aptamer domain folds into an “H-type” pseudoknot motif (42) whereby half of the 5′-helix intercalates between the two halves of the 3′-helix, which contains the embedded RBS (Fig. 2*B*). In most of these pseudoknots, the RBS is directly contacted by the ligand (43, 44, 45, 46), although in the case of the *Thermoanaerobacter tengcongensis (Tte)* preQ_1_-I riboswitch, only the 5′-nucleotide of the RBS is incorporated into the aptamer domain (40). This indicates that there is significant flexibility with respect to the spatial arrangement of the ligand and the RBS and that the occlusion mechanism does not require direct contact between the two. While the H-type pseudoknot is the most common structural motif, other RNA structures can be used to create direct occlusion riboswitches. For example, the aptamer domain of the SAM-III and preQ_1_-II riboswitches comprises a three-way junction motif that hosts the RBS and ligand-binding site (47, 48).

**Figure 2:**
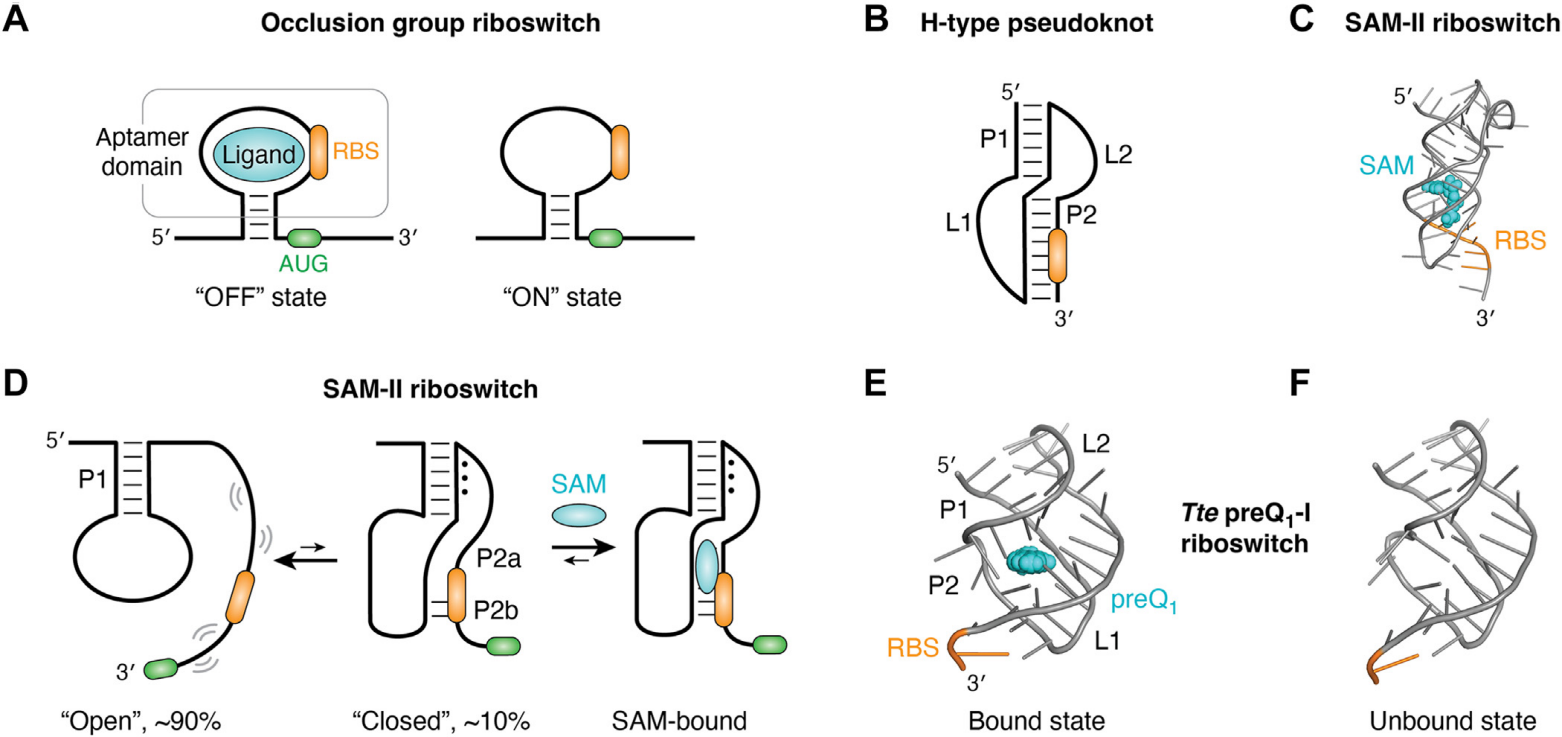
**Examples of direct occlusion group riboswitches.***A*, schematic of a direct occlusion group riboswitch. The aptamer domain contains the information necessary to recruit the ribosome to the mRNA. In the bound state (*left*), ligand binding to the aptamer domain (*gray box*) causes stable incorporation of the RBS (*orange*) into the riboswitch, blocking translational initiation from the AUG start codon (*green*). In the absence of ligand binding (*right*), the RBS is accessible to the ribosome, enabling translation. *B*, secondary structure of an H-type pseudoknot, comprising two coaxially stacked helices (P1 and P2) connected by two linkers (L1 and L2). In occlusion group riboswitches, the RBS is typically embedded in the 3′-helix (P2). *C*, structure (PDB ID 2QWY) of the SAM-II riboswitch with SAM (*cyan*) bound in the major groove of P2 and the RBS directly contacted by the ligand. *D*, mechanism of binding of the SAM-II riboswitch using a conformational selection mechanism, whereby the unbound riboswitch samples an “open” and “closed” state. SAM binds to the closed state, causing further induced fit changes that fully occlude the RBS. *E*, structure (PDB ID 6VUI) of *Tte* preQ_1_-I riboswitch bound to preQ_1_ (*cyan*) and the 5′-nucleotide of the RBS (*orange*). Note that, unlike the SAM-II riboswitch, the RBS is adjacent to, and not fully incorporated into, the ligand-binding element. *F*, structure (PDB ID 6VUH) of the unbound state. The overall structure is very similar to the bound state, maintaining the incorporation of the 5′-nucleotide of the RBS into the pseudoknot fold. RBS, ribosome-binding site.

Understanding the relationship between ligand binding and sequestration of the RBS requires experimental approaches that are able to assess the apo (unbound) state of the RNA, rather than the more easily accessed bound state, as exemplified by the *metX* SAM-II riboswitch (35). Structural analysis of the SAM-bound state by X-ray crystallography revealed the atomic-level details of how the RNA recognizes SAM, coordinates close spatial relationship between SAM and the RBS, and discriminates against the closely related demethylated SAH (43). However, while this structure suggested direct blocking of the RBS embedded within the second helix (P2, Fig. 2*C*), alternative experimental approaches were required to provide information vital to understanding the blocking mechanism.

Generally, examination of the apo state of the RNA requires solution-based approaches that directly interrogate the organization of the aptamer domain in the absence of the ligand. First, the *metX* SAM-II riboswitch was studied using chemical probing approaches (*e.g.*, selective 2′-hydroxyl acylation analyzed by primer extension (49) and dimethyl sulfate (50)) to examine the accessibility of functional groups within the RNA as a function of different conditions (*e.g.*, with and without ligand). Comparison of chemical reactivity patterns of the *metX* SAM-II riboswitch in the absence and presence of SAM revealed that while the P1 helix was protected from chemical modification regardless of the presence of ligand, the P2 helix and its associated L1 element showed SAM-dependent conformational changes. Specifically, P2 and L1 nucleotides were exposed in the absence of SAM but protected in its presence (43). Single-molecule fluorescence resonance energy transfer (smFRET) experiments yielded more quantitative information regarding the presence of distinct exposed and protected states and their rates of interconversion (51). In the absence of Mg^2+^, RNA was predominantly in a low-FRET state, indicative of an “open” conformation where the RNA was in a hairpin configuration with a highly accessible RBS (Fig. 2*D*). In contrast, in the presence of Mg^2+^, a subpopulation of RNA shifted towards a high-FRET state suggestive of a compact, “closed” conformation where part of the P2 helix forms but rapidly interconverts with the open state. Finally, in the presence of Mg^2+^ and SAM, the RNA predominantly exhibits the high-FRET closed state with only transient excursions to the low-FRET open conformation. This model is further supported by small-angle X-ray scattering and solution NMR experiments (52, 53). It is only with these solution-based experiments examining apo and bound forms of the RNA that this three-state model would be conceptualized.

An important question is how the conformational dynamics of SAM-II is coupled to ligand binding. Two potential binding mechanisms are “conformational selection” and “induced fit” (51, 52, 53). The conformational selection mechanism involves an RNA conformational change that occurs prior to binding in which the ligand binds and stabilizes a higher energy and lowly populated RNA excited state. Conversely, in induced fit, the conformational change occurs after ligand binding whereby the ligand induces a conformational change to the ground state RNA. These models of binding represent two extremes along a continuum of binding mechanisms, which is tunable by the relative timescales of RNA conformational transitions and RNA-ligand diffusion (54) and ligand concentration (55). Interestingly, the above data suggest that SAM-II employs both conformational selection and induced fit ligand-binding mechanisms. The conformational selection component of this mechanism is that ligand binding requires an RNA conformational state that is near-native, in which the binding pocket is sufficiently organized for recognition. In SAM-II, this is the interconversion between the hairpin state and the open pseudoknot form (Fig. 2*D*), where only the latter state has sufficient organization of the P2 helix to promote SAM binding. The induced fit component of the binding mechanism is the RNA-RNA and RNA-ligand contacts established after initial ligand docking, yielding the final closed, native state observed in the crystal structure. These data underscore the fact that these binding mechanisms are not mutually exclusive. Indeed, mixed conformational selection/induced fit mechanisms are now considered to be a general model for aptamer domain-ligand recognition within riboswitches (56, 57, 58).

Another example of a well-studied riboswitch using the direct occlusion mechanism is the *Tte* preQ_1_-I riboswitch (40). PreQ_1_ is a biosynthetic precursor to queuosine (Q), a modified nucleotide found in the anticodon loop of tRNA (59). Like SAM, the preQ_1_ riboswitch uses an H-type pseudoknot with the binding pocket embedded within the 3′-helix (P2). However, unlike the *metX* SAM-II riboswitch, the RBS in the *Tte* preQ_1_-I riboswitch is peripheral to the ligand-binding site (orange, Fig. 2*E*) (40). Only the 5′-guanosine of the RBS (5′-GGGAG-3′) is embedded within the aptamer domain at its 3′-end, suggesting that occlusion of this nucleotide alone is sufficient to block ribosome binding. Additionally, further decoupling of the RBS and the ligand-binding site is evident in preQ_1_-II riboswitches. Like the above riboswitches, the core of the preQ_1_-II riboswitch aptamer domain is an H-type pseudoknot, but the second loop of preQ_1_-II riboswitches is interrupted by a stem-loop (41, 60). The ligand binds at the interface between the two pseudoknot helices (P2 and P3), which facilitate the stabile incorporation of the RBS into P3, thereby blocking the ribosome’s access to the message and preventing translation (47, 61). More recently, a novel preQ_1_-I riboswitch that binds two molecules of pre-Q_1_ in the binding pocket (62) is capable of significant translational attenuation when the RBS is removed from the aptamer by up to 10 nucleotides, suggesting that the compact pseudoknot can block ribosomal access at a distance (63). Thus, integration of the aptamer domain and expression platform into a single structural unit may not be an absolute requirement for the direct occlusion group.

Importantly, several crystal structures of the apo state of the *Tte* preQ_1_-I riboswitch have been determined, yielding insights into the conformational state of the RNA recognized by the ligand (64, 65). While crystal structures of aptamer domains in the ligand-bound state are abundant, only a small subset of riboswitches have representative structures of their unbound state (58, 66, 67, 68, 69). The crystal structures of the apo *Tte* preQ_1_-I riboswitch reveal the RNA in a near-native state (Fig. 2*F*), including the incorporation of the 5′-guanosine of the RBS into the aptamer domain (64, 65). These data provide compelling evidence that, even in the absence of ligand, RNA can access compact structures in which the binding pocket is highly organized and poised for ligand binding, in support of conformational selection binding mechanisms. However, crystal structures often favor RNA conformations that optimize lattice interactions but are not well represented in solution—that is, conformationally dynamic states are generally not accessible.

The observation that the unbound and bound aptamer domain structures are very similar in X-ray crystallography structures highlights the need to use solution-based and single-molecule approaches to fully inform the conformational ensemble sampled by the unbound RNA (58), which significantly influences the regulatory decision (27). Using smFRET measurements, it was shown that apo *Tte* preQ_1_-I riboswitch undergoes fluctuations between open and closed states, similar to the *metX* SAM-II riboswitch (Fig. 2) with the closed state poised for ligand binding (70). However, molecular dynamic simulations suggest that ligand binding can occur early in the folding trajectory in conformations where P1 is formed but the 3′-tail has not yet docked to form P2 in the closed state (57, 70). This suggests that binding predominantly follows an induced fit mechanism in which ligand binding promotes the occlusion of the RBS *via* formation of P2. However, as previously mentioned, conformational selection and induced fit mechanisms are not mutually exclusive and individual RNAs can follow paths that are dominated by one or the other.

To further investigate the relationship between ligand binding and RBS accessibility, an innovative single-molecule method called single-molecule kinetic analysis of RNA transient structure was developed (71). This approach uses a fluorescently labeled oligonucleotide antisense to the RBS to observe changes in RBS accessibility as a function of ligand concentration. These data reveal that the RBS in both the apo and preQ_1_-bound states shows stochastic accessibility to the labeled probe, with the bound state showing only a modest ∼two-fold reduction in the frequency at which the probe can access the RBS. This finding suggests that a strong regulatory response may be facilitated by other aspects of mRNA metabolism, such as cotranscriptional folding (72), transcriptional-translational coupling (73), and/or Rho-dependent transcriptional termination in the absence of translation (74, 75). Thus, in the case of some riboswitches, the direct occlusion mechanism may be more complicated than the simple and generalized model illustrated in Figure 2. However, it is highly likely that even in these riboswitches, binding of the effector directly blocks ribosomal access to the mRNA.

Two further points should be made regarding the direct occlusion group of riboswitches. First, an aptamer-defined riboswitch class is not restricted to a single mechanism-based group. An example of this is the *Bacillus subtilis* preQ_1_-I riboswitch (39, 76). This riboswitch, along with related preQ_1_-I riboswitches in Firmicutes, regulates transcription through the formation of an intrinsic terminator in a ligand-dependent manner using a strand exchange mechanism (see below) (39, 77, 78). However, *Tte* preQ_1_-I riboswitch discussed above uses the direct occlusion mechanism. This emphasizes that a riboswitch classified according to the identity of the ligand they bind and/or the secondary/tertiary structure of their aptamer domain may belong to multiple groups based on regulatory mechanism of the expression platform. Second, while the above examples highlight how ligand-binding blocks translation, the opposite “unblocking” mechanism has been proposed for the guanidine-II riboswitch (79). Like their blocking counterparts, these regulatory elements are translational switches where the RBS is stably incorporated into the apo state of the aptamer domain, while ligand binding liberates the RBS (80, 81).

### Group 2: Interdomain docking

The docking regulatory mechanism differs from the occlusion mechanism in that productive ligand binding does not require a single integrated riboswitch unit—these RNA elements have a distinct and separable aptamer domain and expression platform (Fig. 3*A*). In this riboswitch group, the aptamer domain is capable of independently binding a specific ligand (Fig. 3*A*) but does not contain a sequence element capable of directly communicating with the expression machinery. The aptamer domains within this group are more structurally complex than their occlusion group counterparts, likely reflecting a more complicated mechanism for coupling ligand binding to regulatory activity. Ligand binding to the aptamer domain promotes interactions between the aptamer domain and the downstream expression platform. In the case of translational control, the interaction of the aptamer domain-ligand complex with the expression platform occludes the RBS from the ribosome, thereby repressing translation (Fig. 3*A*). However, in the absence of ligand binding, the RBS is accessible for translation. Examples of riboswitch classes that use this organization to regulate gene expression are preQ_1_-III (60), cobalamin (Cbl) (6), cyclic-di-adenosine (*ydaO*) (82), and azaaromatic (*yjdF*) (83) riboswitches.

**Figure 3:**
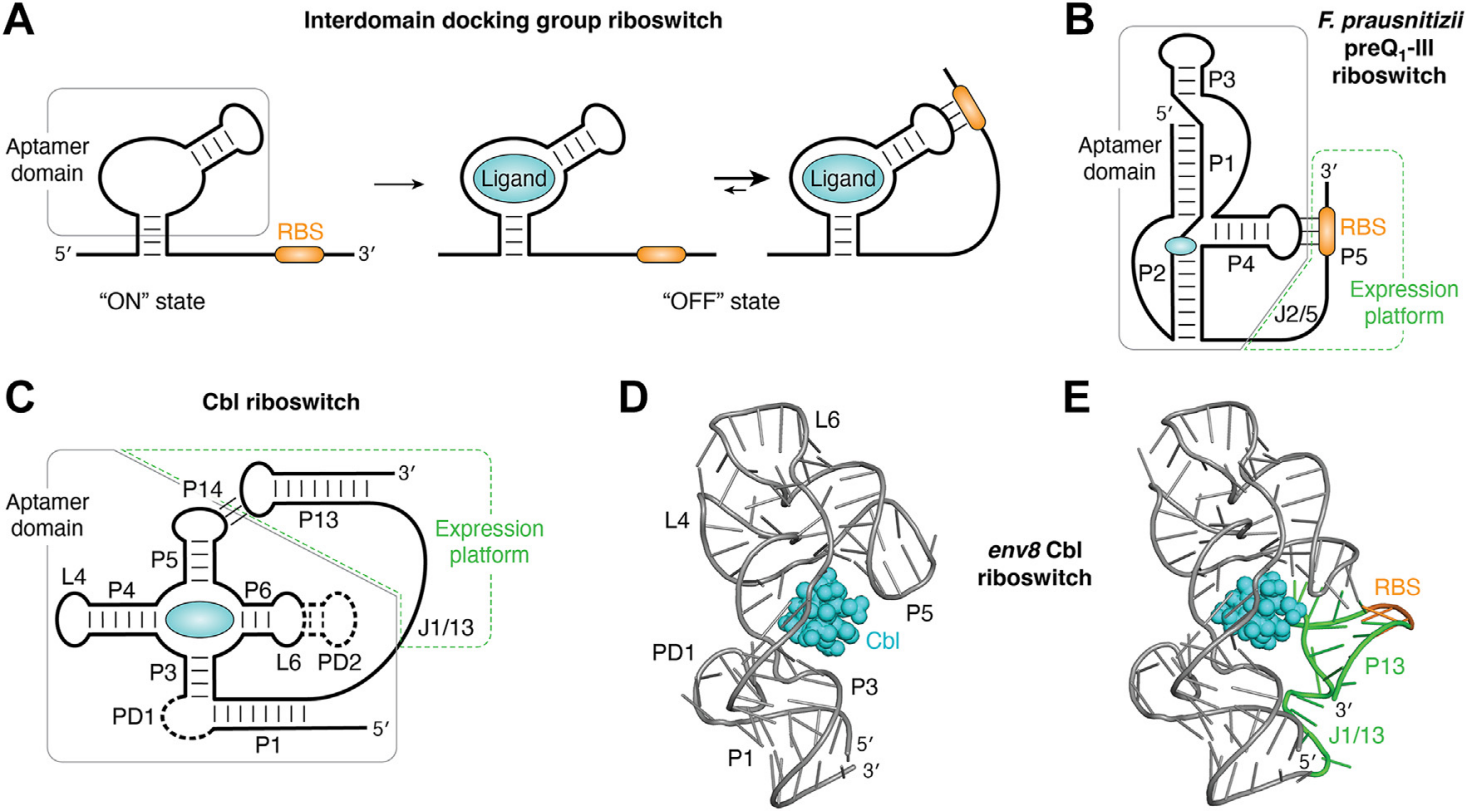
**Example of interdomain docking group riboswitches.***A*, the aptamer domain (*gray box*), defined as the minimal RNA sequence capable of full affinity ligand binding, does not contain information that informs the expression machinery (*left*). A key characteristic of this riboswitch group is that their aptamer domain is capable of independently binding the ligand (*center*). Ligand (*cyan*) binding promotes the recruitment of the expression platform to the aptamer domain to direct the expression machinery (*right*). In this example, the translation of the message depends on the accessibility of the ribosome to the RBS (*orange*) within the expression platform. *B*, schematic of the secondary structure of the *F. prausnitizii* preQ_1_-III riboswitch in the bound state. The aptamer domain, which can bind preQ_1_ independently of the expression platform, is denoted by a *gray box*, while the expression platform, containing the interdomain linker and RBS (*orange*), is denoted by a *dashed green box*. Docking of the expression platform with the aptamer domain creates helix P5, which occludes the RBS from the ribosome. *C*, schematic of the conserved core of Cbl riboswitches. The expression platform (*dashed green box*) consists of a hairpin whose loop docks with a loop in the aptamer domain (*gray box*) to form a new helix. Formation of this interdomain interaction informs the expression machinery. *D*, structure (PDB ID 4FRG) of the *env*8 Cbl riboswitch aptamer domain in complex with hydroxocobalamin (*cyan*). *E*, structure (PDB ID 4FRN) of the full-length *env8* Cbl riboswitch, with the expression platform highlighted in *green* and the RBS highlighted in *orange*. Note that the aptamer domain is nearly identical in both structures, with the ligand facilitating docking of the expression platform with the aptamer domain. RBS, ribosome-binding site.

PreQ_1_-III riboswitches are significantly larger than their class I and II counterparts (60), reflecting a more complex “HL_out_” type pseudoknot (Fig. 3*B*) (84), which is structurally similar to the H-type pseudoknots. The crystal structure of the *Faecalibacterium prausnitizii* (*Fpr*) preQ_1_-III riboswitch reveals a global architecture in which the ligand is embedded within the interhelical junction between P1, P2, and P4 (85). Analysis of ligand binding to the *Fpr* preQ_1_-III riboswitch and a second representative indicate that full affinity binding is supported by a core aptamer domain spanning P1-P4 (60, 85), which is likely a general property of this riboswitch class. However, sequence analysis reveals that the RBS is downstream of P4 and adjacent to a sequence that forms a second pseudoknot by base pairing with the terminal loop of P4 to form the P5 helix (85). smFRET experiments revealed that preQ_1_ binding only marginally stabilizes the P5 helix, which sequesters the RBS to repress translation. Instead, ligand binding increases the population of RNAs that have P4 positioned at an acute angle relative to the P2-P1-P3 coaxial stack (Fig. 3*B*), enabling docking of the expression platform with the terminal loop of P4 to form P5. Given the modest effect of ligand binding on stabilizing the sequestered state of the RBS, this riboswitch may use other means to elicit the full biological regulatory response (71).

A second well-studied example of ligand-mediated docking of the expression platform with the aptamer domain is the Cbl riboswitch (86). These are some of the most broadly distributed riboswitches, found in every major clade of bacteria, suggesting that this riboswitch is a genetically and physiologically flexible mechanism for regulating the uptake and synthesis of cobalamins (6, 7, 8). The general architecture of Cbl riboswitches is a four-way junction between helices P3-P6 that is organized by a conserved T-loop-mediated tertiary interaction between L4 and L6 (Fig. 3*C*) (87). Cbl mainly interacts with the four-way junction, with two peripheral domains (PD1 and PD2) mediating selectivity of the RNA for one of the two biological forms of Cbl: adenosylcobalamin and methylcobalamin (Fig. 3*C*) (88). Like the preQ_1_-III riboswitch, the aptamer domain alone is capable of high affinity ligand binding (88). Crystal structures of the aptamer domain and full-length *env8* (the “*env*” designation is for environmental sequence derived from a metagenome) Cbl riboswitch bound to hydroxocobalamin reveal almost identical structures, further demonstrating that the expression platform is not necessary for ligand binding (Fig. 3*D*). Superimposition of the bound structures of the aptamer domain (89) and full-length (90, 91) cyclic-di-adenosine 5′-monophosphate riboswitch show the same feature. In the *env8* Cbl riboswitch that represses translation in response to ligand binding, the expression platform consists of a small hairpin (P13) containing the RBS in its terminal loop (88). In the riboswitch-Cbl complex, this loop forms a “kissing loop” interaction with the terminal loop of P5 in the aptamer domain to form the P14 helix that occludes the RBS from the ribosome. Reinforced by smFRET experiments, a general model has emerged in which ligand binding at the aptamer domain promotes the docking of the expression platform to the aptamer domain–ligand complex, forming the translationally repressed state (92). Unlike the preQ_1_-III riboswitch, Cbl binding dramatically stabilizes formation of the RBS-occluded state, with undocking events being rarely observed in the Cbl-bound form.

A key question is how the Cbl riboswitch harnesses the L5-L13 kissing loop interaction for ligand-dependent regulation of mRNA expression. The kissing loop is a widespread module observed in diverse biological RNAs that promote higher-order RNA structure and *trans* RNA-RNA interactions (93). Typically, these interactions involve between five and seven Watson-Crick base pairs between two terminal hairpin loops to create a new helical element (such as P14, Fig. 3*C*) that stacks between the stems of the two interacting hairpins (94). Generally, this interaction is stable and forms in the absence of other factors (95, 96, 97), although proteins (98, 99) or small molecules (79, 81, 100) have been shown to facilitate this interaction. To examine the Cbl-dependence of this interaction, a *trans* aptamer/expression platform was created in which Cbl-dependent binding of the expression platform hairpin was monitored using an EMSA (101). A detailed mutagenic survey of the loop-loop interaction revealed that the key structural feature conferring Cbl-dependent kissing loop formation was a one or two nucleotide bulge in the interloop helix (P14, Fig. 3*C*), consistent with the finding that non-Watson-Crick pairs in the interloop helix destabilize the interaction (102). Examination of the crystal structure reveals that P13 is directly contacted by the ligand (Fig. 3*E*), suggesting that interdomain interaction and thereby occlusion of the RBS is achieved through a combination of long-range RNA-RNA and RNA-small molecule interactions (88). Together, these data suggest that Cbl riboswitches use a destabilized tertiary interaction to mediate interdomain docking where the ligand directly provides a set of contacts that stabilize docking to occlude the RBS from the mRNA.

### Group 3: Strand exchange

Strand exchange riboswitches employ a secondary structural switch to produce two distinct and mutually exclusive regulatory states, “ON” or “OFF” (Fig. 1) (3). Strand exchange (also known as strand displacement) is the most common mechanism used to create a binary “switch” that generates two distinct states of expression, and it can be applied to control transcription (Fig. 1*A*) or translation (Fig. 1*B*). Like the interdomain docking group, strand exchange riboswitches contain a distinct aptamer domain and expression platform. At the heart of this mechanism is the switching sequence, a segment of RNA that forms one of two mutually exclusive secondary structures corresponding to the expression “ON” and “OFF” states (red, Fig. 1) (25). In one state, the switching sequence, most commonly found in the first helix, (Fig. 1), is incorporated into the aptamer domain, creating a state that supports ligand occupancy. This fates the downstream expression platform to form a secondary structure without the switching sequence. In the absence of ligand binding, the switching sequence can be incorporated into the expression platform to create an alternative secondary structure that changes the instruction to the expression machinery (Fig. 1). The key feature of the strand exchange group that differentiates it from the occlusion and docking groups is the disruption of the unbound–but ligand-binding competent–state of the aptamer domain by the expression platform.

Strand exchange is a process in which a single-stranded nucleic acid hybridizes to a duplex DNA or RNA target, displacing one of the original duplex strands *via* branch migration (103, 104). For this to occur, the invading sequence (blue strand, Fig. 4) initiates the process by interacting with a single-stranded region adjacent to the duplex that will be disrupted. The single-stranded region is called a “toehold” and serves as a site for nucleation of the new helix. Then, the single strand displaces its counterpart from the duplex to form a new duplex (Fig. 4). Importantly, displacement is bidirectional except for the last step, which is irreversible due to the lack of a toehold for the displaced strand to re-invade. In synthetic biology, this simple mechanism has been used to create a range of nucleic acid devices, generally called “toehold switches” (105, 106, 107). Riboswitches harness this process by using ligand binding to the aptamer domain to either directly or indirectly block the exchange process by stabilizing the helix to be invaded (108, 109).

**Figure 4:**
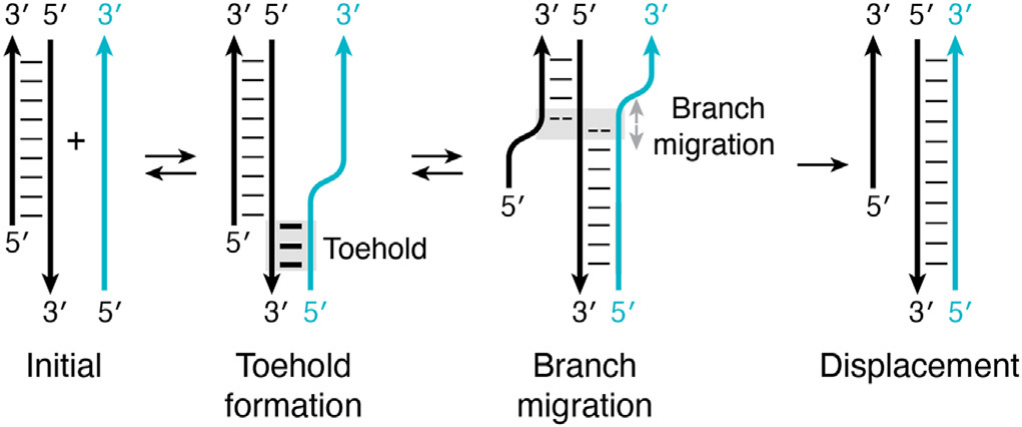
**Schematic of the strand exchange process.** The invading sequence (*blue* strand) initiates strand exchange by interacting with a single-stranded region (toehold) adjacent to the duplex that will be disrupted. Toehold formation then nucleates the formation of a new helix, which after a branch migration event results in complete displacement. All steps in this mechanism are reversible with the exception of the final step. This irreversibility is due to the lack of a toehold for the displaced strand, rendering it unable to initiate a new displacement reaction.

In contrast to the occlusion and docking groups, which almost exclusively regulate gene expression by manipulating an RBS, strand exchange riboswitches can readily interface with different expression machineries. The thiamine pyrophosphate (TPP) class of riboswitches (5, 110), the most broadly distributed across biology (7, 8), is an outstanding example of an aptamer domain’s ability to inform diverse expression machineries using strand exchange. For example, in bacteria, TPP riboswitches instruct RNAP *via* ligand-dependent formation of an intrinsic terminator (111), the ribosome through exposure or occlusion of the RBS (110, 112, 113), and dual forms of regulation where the aptamer domain directly informs translation directly *via* the RBS and indirectly *via* Rho-dependent transcriptional termination (114). In fungi, the TPP riboswitch manipulates the accessibility of an alternative splice site and branch site of an intron. In *Neurospora crassa*, splicing of the *NMT1* and *NCU01977* genes are repressed in the absence of TPP *via* regulation of the accessibility of the 5′-splice site of the intron where the aptamer is embedded (115, 116). Finally, while it has not been observed for TPP riboswitches, other classes of riboswitches use strand exchange to recruit or block RNases that degrade the message (117) or a Rho utilization (rut) site to terminate transcription (75).

As described above, direct occlusion and interdomain docking riboswitches have yielded mechanistic insights into the relationship between ligand binding and the regulatory response. In contrast, obtaining this level of detailed understanding for strand exchange riboswitches has been much more difficult. A major factor in this deficiency is that full-length strand exchange riboswitches have not been amenable to structural analyses that would generate testable hypotheses regarding structure-function relationships. To date, there is no high-resolution structure of a complete strand exchange riboswitch, although advances in cryo-EM of smaller RNAs are changing this (118, 119, 120). For example, the Walter group has recently described the cryo-EM structure of the *B. subtilis* pre-Q_1_ riboswitch bound to RNAP in the ligand-free and ligand-bound states to reveal how RNA structure in the nascent transcript interacts with the transcriptional machinery (121). Additionally, regulation by the nascent transcript has been greatly informed by cryo-EM structures that provide a series of snapshots that reveal the structural basis of intrinsic termination (122).

Over the last few years, an increasing number of biochemical and biophysical studies have proposed detailed cotranscriptional models of ligand-dependent regulation by riboswitches. Such studies include adenine (28), guanine (123), 2′-deoxyguanosine (124), preQ_1_ (29), fluoride (125), TPP (126), three 5-aminoimidazole-4-carboxamide riboside 5′-monophosphate (ZMP, which is an intermediate in purine biosynthesis) (109, 127, 128), and cyclic-di-guanosine 5′-monophosphate (c-di-GMP) and cyclic guanosine-adenosine 5′-monophosphate (129) riboswitches. It is important to note that each of these studies used a different set of experimental approaches to develop models that account for aptamer domain folding, ligand binding, and secondary structural exchange as a function of transcription progression. Consequently, these models have different levels of detail regarding folding and reaction kinetics, nucleotide-resolution folding progress, considerations of pausing, and potential for regulatory proteins. Despite their differences, each model makes important contributions towards the development of a comprehensive and unified understanding of how riboswitches make critical decisions as transcription progresses to elicit the appropriate regulatory response.

For transcriptional riboswitches that manipulate formation of an intrinsic terminator, a regulatory decision must be made while RNAP is actively transcribing the poly-uridine track immediately downstream of the hairpin and thus operate under temporal constraints (31, 32). In this mechanism, the intrinsic terminator forms in the RNA exit channel of RNAP and causes the mRNA to disengage through shearing of the RNA-DNA hybrid within the transcription bubble (122, 130). Once RNAP proceeds beyond the terminator hairpin, the riboswitch can no longer govern mRNA synthesis. Due to the coupling of transcription and translation (131, 132, 133) in many bacteria, such as the Proteobacteria which tend to use riboswitches that control translation (7), the majority of translational riboswitches can only function in the context of transcription. The kinetic features of RNA folding, ligand binding, and strand exchange govern the regulatory response by a process known as “kinetic control”, a feature of riboswitches that was recognized soon after their discovery (134, 135). Kinetic control is a process in which there is insufficient time for the system to reach full equilibrium and thus the outcome is dictated by the rates of the reaction. The observation that most strand exchange riboswitches only function in the context of transcription is a significant obstacle to developing a comprehensive understanding of their regulatory mechanism due to the lack of *in vitro* experimental approaches that authentically recapitulate transcription in the cellular environment. Despite this limitation, there are an increasing number of studies, some discussed below, using creative approaches to circumvent this constraint to develop mechanistic models of regulation *via* ligand-dependent strand exchange.

For example, one study used cotranscriptional selective 2′-hydroxyl acylation analyzed by primer extension-seq to investigate the cotranscriptional folding of the *Clostridium beijerinckii pfl* ZMP riboswitch (109). This approach uses chemical probing of a set of RNAs that recapitulate the stepwise addition of a single nucleotide to the nascent RNA chain during transcription, either in the absence or presence of the ligand. In the model proposed by this study, the earliest stages of the folding pathway involve formation of non-native (IH1) and native (P2) hairpins (“IH folded” and “P2 folded”, Fig. 5). A non-native secondary structure in the aptamer domain was also proposed to be important for temperature-dependent regulatory activity of the *Vibrio vulnificus add* adenine-responsive riboswitch, suggesting that these structures may also play an important role in regulating RNA folding (136). Further stages of aptamer domain folding involve the remodeling of IH1 into P1 as the 3′-end of P1 is synthesized and formation of P2 and P3 (“P1 folded” to “unstable aptamer fold”, Fig. 5). Critical to the formation of P3 is a long-range pseudoknot between J1/2 and L3 that is required to form the ZMP-binding pocket, as observed in crystal structures of the ZMP-bound aptamer domain (137, 138, 139). At this stage of the folding pathway (“unstable aptamer fold”, Fig. 5), the riboswitch is competent for ligand binding since all necessary and sufficient sequence elements have been transcribed (140). An important caveat of this model is that since ∼14 nucleotides of the nascent transcript remain within the RNAP (141, 142) and therefore are inaccessible for folding, this point actually represents mRNA synthesis beyond the 3′-end of the P3 helix. To increase the window of time where the aptamer domain can bind ZMP prior to the expression platform disrupting the binding-competent structure, there are two pause sites ∼20 nucleotides downstream of the 3′-end of the P3 helix (109). It is important to note that this mechanism requires that the aptamer domains of strand exchange riboswitches have the ability to rapidly acquire their proper secondary and tertiary structure with high fidelity, evading kinetic folding traps and unproductive alternative secondary structures that plague other RNAs.

**Figure 5:**
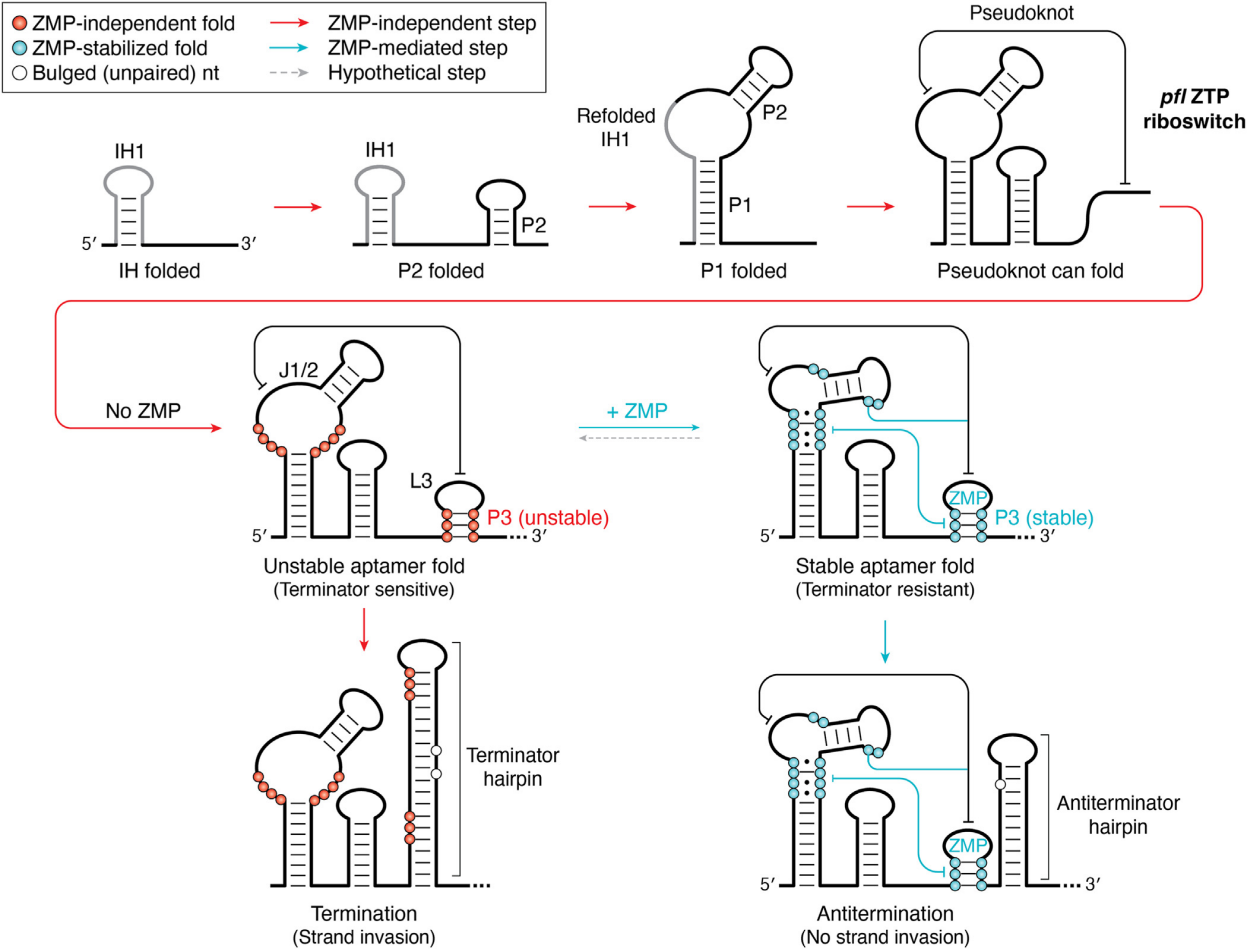
**Cotranscriptional folding model of the *pfl* ZTP riboswitch.** Initial aptamer domain folding involves the transient formation of IH1 and the folding of P2. IH1 is then remodeled into P1 and the 3′-end of P1 is synthesized to form P3. A critical component of P3 formation is a long-range pseudoknot that is required to form the ZMP-binding pocket. With these sequence elements in place, the riboswitch is competent for ligand binding. In the absence of ZMP, an unstable aptamer domain fold results in the sequential disruption of P3 and the pseudoknot to form a terminator hairpin, terminating transcription *via* a strand exchange mechanism. In the presence of ZMP, a stable aptamer domain fold renders the P3 stem resistant to terminator formation, driving antitermination without strand exchange. This model is based on (109).

At the point where the riboswitch can begin to interrogate the cellular environment for their ligand, the transcriptional reaction coordinate bifurcates into two folding pathways that yield the alternate regulatory outcomes (“termination” and “antitermination”, Fig. 5) (109). In one pathway, ZMP binds the aptamer domain to promote a set of interactions between P1 and P3 that stabilize the aptamer domain, preventing invasion from the expression platform (blue lines in antitermination state, Fig, 5). This causes the mRNA to form an antiterminator hairpin, enabling RNAP to proceed past the riboswitch and synthesize the complete message. However, in the absence of ZMP binding, nucleotides downstream of P3 in the expression platform can invade into the stem of P3 to yield the terminator hairpin (“termination”, Fig. 5). Interestingly, the intrinsic stability of the P3 helix was found to be critical for the ability of the expression platform to initiate strand exchange, with the nucleating base pairs favoring the formation of the alternative terminator. Strand exchange proceeds through the entirety of P3, disrupting the long-range pseudoknot between L3 and J1/2 that formed during aptamer domain folding to create the classic Rho-independent (intrinsic) transcriptional terminator and causing RNAP to abort. No RNAP pause sites are observed within the expression platform, suggesting that the kinetics of terminator hairpin nucleation and propagation of strand exchange is very rapid. Given that there are only ∼20 nucleotides between the region that disrupts the base of P3 and the poly-uridine tract of the terminator and assuming an average transcription rate of ∼50 nucleotides/s (143, 144), the strand exchange process has ∼400 milliseconds to complete the formation of the terminator hairpin that is required for efficient termination–consistent with the proposed rates of conformational switching (145).

A second example of a cotranscriptional riboswitch folding model is from a study of *Clostridium difficile* Cd1 c-di-GMP and *G. metallireducens pilM* cyclic guanosine-adenosine 5′-monophosphate riboswitches (129). In this study, solution NMR and isothermal titration calorimetry were used to determine the minimum and maximum lengths of RNA transcripts with ligand-binding capability within the regulatory window set by ongoing transcription. Then, building off previous computational modeling of the *B. subtilis xpt-pbuX* guanine riboswitch (123), isothermal titration calorimetry-derived kinetic rates, and previously determined nucleobase closing rates (146) were used to model riboswitch function. These models showed high sensitivity to ligand concentration, with very low populations of the ligand-bound RNA conformation at low ligand concentrations and very high populations at increased ligand concentrations (129). Moreover, transcription speed had a robust effect on the regulatory function of both riboswitches, always favoring the ligand-bound state at slow speeds. This model is consistent with the idea that slower transcription speeds allow for a longer presence of binding competent states, increasing the likelihood of forming a stable ligand-bound RNA complex (147). Conversely, at faster transcription speeds, switching efficiency was greatly reduced. For example, the *C*. *difficile* Cd1 c-di-GMP riboswitch showed lower sensitivity to fast transcription speeds, likely due to its longer expression platform prior to the point where the regulatory decision must be made (129). Addition of a 10 s pause delay in the expression platform resulted in a switching efficiency of 95% over a window of 5 to 100 nucleotides/s, making it practically insensitive to transcription speed, reinforcing the role of RNAP pausing to modulate the kinetic features of riboswitch function.

## Riboswitches that do not easily fall into the above scheme

The above classification of riboswitch-mediated regulatory mechanisms does not readily accommodate all of the known riboswitches. One such example is the glucosamine-6-phosphate–responsive riboswitch/ribozyme that is found in the 5′-leader of the glutamine-fructose-6-phosphate amidotransferase (*glmS*) gene of numerous Gram-positive bacteria (148). Like members of the occlusion group (Fig. 2), structural analysis of this RNA suggests a single domain (67) in which glucosamine-6-phosphate-mediated strand scission promotes the degradation of the message by providing a 5′-hydroxyl group for RNase J1 (148, 149). While the *glmS* ribozyme was originally classified as a riboswitch on the basis of its binding and utilization of a small molecule (148), this RNA does not instruct the expression machinery like traditional riboswitches. Given this fact, we argue that the *glmS* RNA is a metabolite-responsive ribozyme, not a riboswitch. While it is possible that ligand-induced catalysis and recruitment of strand scission machinery is a general regulatory mechanism that warrants its own unique group, more biological examples are needed.

Another example of difficult-to-classify riboswitches are the T-boxes, which are broadly distributed bacterial mRNA regulatory elements that directly bind specific tRNAs (150, 151). Like members of the interdomain docking group (Fig. 3), T-box aptamer domains independently bind their tRNA ligand, presenting the aminoacyl acceptor arm of tRNA to the downstream expression platform to direct the regulatory response (152, 153). Within the T-box class of riboswitches are distinct subclasses that control transcription (152, 153) or translation (154). For both subclasses, the tRNA’s aminoacyl acceptor arm makes no direct contacts to the aptamer domain and thus tRNA binding to the aptamer domain is independent of aminoacylation status (151). Instead, the aminoacyl acceptor arm docks into the expression platform, which assesses the aminoacylation status and regulates the message using two distinct mechanisms.

**Figure 6:**
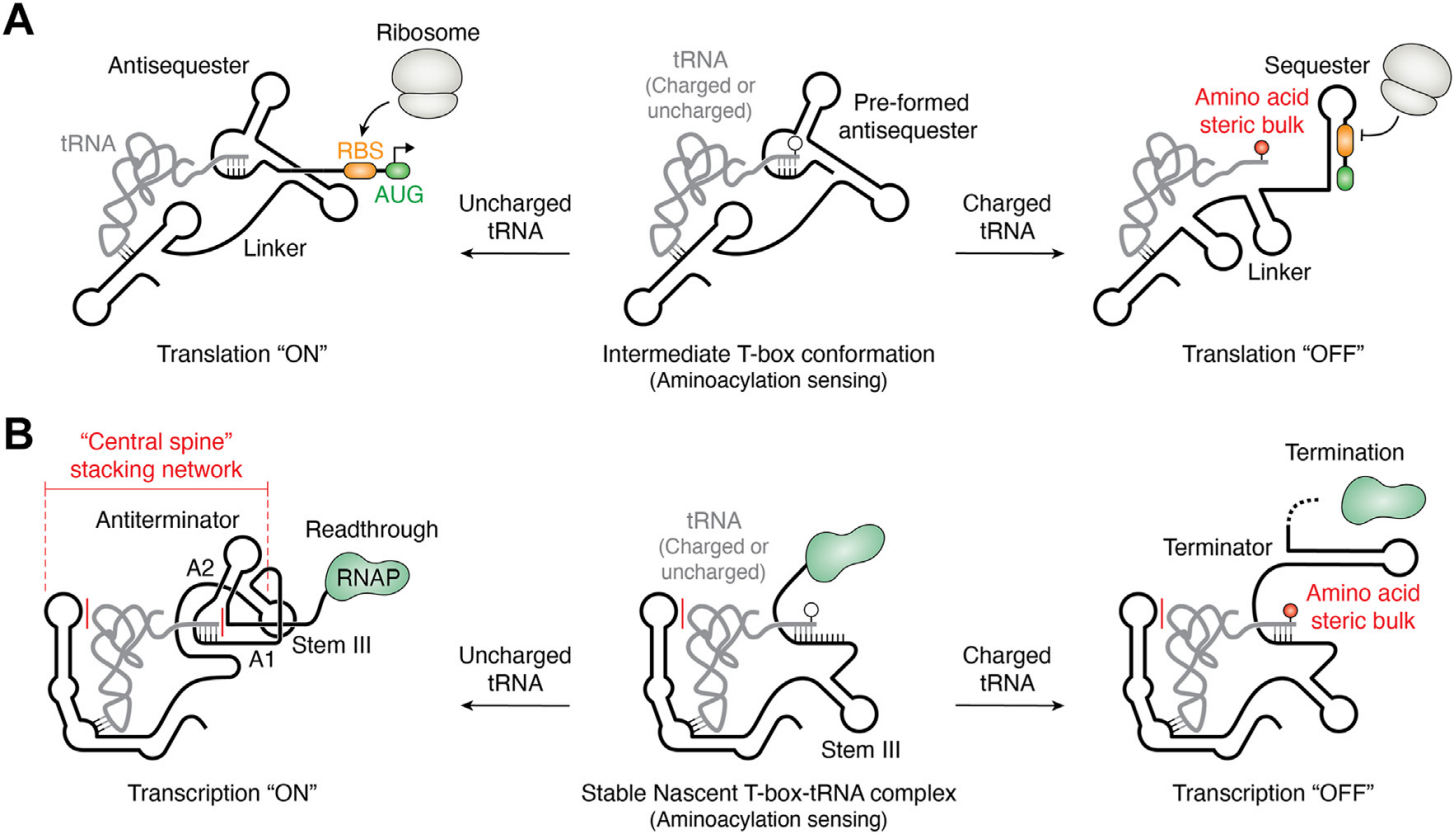
**Schematic of T-box riboswitch regulatory mechanisms.***A*, schematic of translational regulation by the *Mtb-ileS* T-box (154). Aminoacylation sensing (*center*) occurs through an RNA intermediate with a pre-formed antisequester hairpin that is capable of binding both charged and uncharged tRNA. In the presence of uncharged tRNA (*left*), the pre-formed antisequester hairpin remains, permitting access of the ribosome to the RBS to initiate translation. In the presence of charged (aminoacylated) tRNA (*right*), the tRNA 3′-end imposes a steric clash that unravels the linker region, leading to the occlusion of the RBS (*orange*) and AUG start codon (*green*) within a sequester hairpin, preventing translation initiation. Interestingly, the expression platform uses strand invasion to form the terminator hairpin, suggesting that the *Mtb-ileS* T-box employs two distinct mechanisms to regulate translation. *B*, schematic of cotranscriptional regulation as exemplified by the *B. subtilis glyQS* T-box (155). Aminoacylation sensing (*center*) occurs through a stable nascent T-box-tRNA intermediate that is still being actively transcribed. In the presence of uncharged tRNA (*left*), RNA folding is unperturbed by the tRNA 3′-end, leading to the formation of the “central spine” stacking network that stabilizes an antiterminator hairpin, enabling transcription readthrough. In the presence of charged tRNA (*right*), the tRNA 3′-end imposes a steric clash that disrupts the positioning of the central spine, leading to the formation of a terminator hairpin and prematurely terminating transcription. RBS, ribosome-binding site.

One model of how the expression platform interrogates the aminoacylation status of the bound tRNA is exemplified by the *Mycobacterium tuberculosis* isoleucine *Mtb-ileS* T-box that regulates translation (154). Here, aminoacylation sensing occurs through an intermediate RNA conformation that can productively bind both versions (*i.e.*, charged and uncharged) of its tRNA ligand (154). Interestingly, this intermediate has a pre-formed antisequester hairpin (Fig. 6*A*). In the presence of uncharged tRNA, the antisequester hairpin-containing T-box complex is unperturbed, enabling the ribosome access to the RBS to initiate translation (Fig. 6*A*) (154). Alternatively, owing to the increase in steric bulk at the tRNA 3′-end, interaction with charged tRNA dislodges the linker region, leading to conformational changes that favor the formation of a sequester hairpin that blocks ribosome access to the RBS, preventing translation initiation (Fig. 6*A*) (154). Interestingly, the expression platform uses strand invasion to promote terminator formation, suggesting that our mechanism-based classification scheme is not mutually exclusive.

In contrast to the *Mtb-ileS* T-box example above, transcriptional T-boxes initially bind their tRNAs cotranscriptionally and bifurcate into two mutually exclusive RNA conformations depending on aminoacylation status. In the presence of already docked uncharged tRNA, folding between helices A1 and A2 and stem III are unperturbed, leading to the formation of the “central spine” stacking network that stabilizes an antiterminator hairpin, enabling transcription readthrough (Fig. 6*B*) (155). Alternatively, in the presence of already docked charged tRNA, the steric bulk at the tRNA 3′-end prevents the proper folding of helices A1 and A2, which disrupts the positioning of stem III and the entire stem I-tRNA-stem III stacking network (Fig. 6*B*) (155). This lack of stabilization promotes the 3′-strands of helices A1 and A2 to form the 5′-strand of a terminator hairpin that prematurely halts active RNAP transcription (Fig. 6*B*) (155). Together, these examples illustrate that T-box riboswitches use a combination of mechanisms to regulate gene expression.

## Can this conceptual framework be translated into a robust riboswitch classification scheme?

The above framework enables riboswitches to be classified into a small set of groups based up their regulatory mechanism—as opposed to the numerous aptamer-based classes—that encompasses most riboswitches and could potentially include other forms of regulation *via* condition-dependent RNA switching such as thermosensors (156, 157). However, a drawback of this framework is its dependence on experimental analysis to reveal functional sequence features in the expression platform. Robust bioinformatic annotation of expression platforms would enable this framework to be translated into a useful classification scheme, providing a valuable resource to basic research and practical applications.

Current bioinformatic pipelines for riboswitch discovery and classification focus on aptamer domain sequence and structure conservation patterns. In a commonly used resource for RNA sequence information and alignments, Rfam, the expression platform is excluded from most of the riboswitch families’ sequence alignments (158, 159, 160). For example, the purine riboswitch family (RF00167) seed alignment contains the entire aptamer domain and ∼15 nucleotides on its 5′- and 3′-ends, which is generally insufficient to fully comprise the expression platform of purine riboswitches. To be truly useful to the experimentalist, alignments across the entire riboswitch sequence must endeavor to fully annotate sequence features of the expression platform critical for gene regulation.

For riboswitches acting at the level of transcription, the most easily identifiable regulatory element is an intrinsic (or Rho-independent) transcriptional terminator. This element is a GC-rich region that forms a stem-loop with a length of at least eight base pairs and is followed by a poly-uridine tract that serves to pause RNAP and promote the dissociation of the mRNA transcript from template DNA (31, 32). While this signal is broadly distributed across bacteria, there are variants that lack the canonical terminator, which may confound their identification (161, 162). The alternative mechanism to intrinsic termination is Rho-dependent termination, where the Rho hexamer binds to a rut site that tends to be unstructured and rich in cytidine nucleotides. While Rho is essential in *Escherichia coli*, it is not essential in other bacteria such as *B. subtilis*. Moreover, although rut sites can be bioinformatically determined, they reside within the ORF in some riboswitches (74), which can complicate assessing whether Rho-dependent termination is a component of the regulatory mechanism in the absence of experimental validation.

For riboswitches acting at the level of translation, the expression platform almost invariably contains the mRNA’s RBS. The bacterial RBS is typically purine-rich and base pairs with the 16*S* rRNA of the 30*S* ribosomal subunit during translation initiation (163, 164). While the RBS is a slight variation of the 5′-GGAGG-3′ core in most bacteria, the observed sequence and degree of usage varies. For example, in *E. coli*, many genes do not contain a recognizable RBS sequence (165, 166) and instead rely on other mechanisms of positioning the ribosome upstream of the initiating codon such as ribosomal protein S1 (167). These sites may be more difficult to identify using bioinformatic approaches.

Another useful annotation site within the expression platform is a “programmed pause” that causes RNAP to temporarily stall. Uridine-rich pause sites, originally identified in the *B. subtilis ribD* Flavin mononucleotide (134) and *pbuE* adenine (135, 168) riboswitches, revealed that the timeframe of transcription is crucial for eliciting the appropriate regulatory response. In these classic studies, it was found that without RNAP pausing, the rate of transcription is sufficiently rapid that RNAP could easily clear the terminator region before the aptamer domain has had sufficient time for ligand binding. Uridine-tracts within the expression platform pause RNAP on the second-to-minute timescale to provide the riboswitch with additional time for aptamer domain folding, ligand binding, and secondary structural switching. In agreement with this phenomenon, experimental interventions that increase the time spent at pause sites, such as the addition of the ubiquitous bacterial transcription elongation factor NusA or decreasing ribonucleotide concentrations, also influence regulatory switching efficiency (147, 168). The observation that, despite the pause, the aptamer domain does not fully equilibrate prior to the regulatory decision means that the rates of transcription, RNA folding, ligand association/dissociation, and structural switching dictate the regulatory response, not just the intracellular concentration of ligand. In other words, these processes are under kinetic rather than thermodynamic control. Consequently, the identification and annotation of RNAP pause sequences is critical to advancing our understanding of how riboswitches control gene expression.

Currently, there are several classes of pause sequences that have been identified beyond the uridine-tract. For example, the “elemental” pause has a consensus sequence of 5′-G_-11_G_-10_/Y_-1_G_+1_-3′ and was identified using native elongating transcript sequencing approaches in *E. coli* and *B. subtilis* (169, 170, 171). However, there may be pauses that are unique to specific riboswitch classes or RNAs within a given class, such as the “que pause”, which was recently found in the *B. subtilis queC* preQ_1_-III riboswitch, which regulates transcription (29). In this type of pause site, it was observed that the riboswitch’s secondary structure had a significant influence on the lifetime of RNAP pausing. Recent cryo-EM structures of the *queC* preQ_1_-III in the free and bound state support the idea that folding of the nascent transcript and RNAP influence one another (121). In the absence of preQ_1_, the nascent transcript is sterically hindered, preventing downstream nucleotide incorporation. However, upon ligand binding, the riboswitch undergoes a conformational change to release the transcriptional pause. As revealed by molecular dynamic flexible fitting analysis, preQ_1_ binding induces the riboswitch to rotate around its helical axis, widening the RNAP exit channel to provide a clear path for the nascent transcript. Finally, specific factors can have variable effects on a pause sequence. For example, the ubiquitous general transcription elongation factor NusG positively influences pausing in *B. subtilis* (172) while diminishing pausing in *E. coli* (173). Given that riboswitches are often studied in heterologous systems (*i.e.*, a *B. subtilis* riboswitch assessed using *E. coli* RNAP *in vitro* or used in a cell-based reporter in *E. coli*), our understanding of the riboswitch’s function in its native genetic context is limited.

In all of the above examples, the sequence and/or structural element(s) are used to recruit or act on the expression machinery, but there are other expression platform elements to act on. Not all the information within the expression platform is directly related to instructing the expression machinery. Instead, many expression platforms contain secondary structural elements that reinforce efficient regulation without comprising the core regulatory switch. An example of this is found in the *E. coli lysC* riboswitch expression platform, which contains a hairpin element that is found within the bound and unbound states, independent of ligand binding (174). This element was found to positively influence the formation of the antiterminator hairpin in the unbound “ON” state. The *B. subtilis pbuE* adenine riboswitch expression platform also contains a hairpin structure important for nucleation of the terminator helix (175, 176). Furthermore, T-box riboswitches have a pseudoknot in the expression platform required to elicit the aminoacylation-tRNA-dependent response, despite not being involved in the regulatory switch (150). These types of elements are generally overlooked but can also harbor functional information that is crucial for efficient RNA folding or formation of alternative structures. Thus, annotation of secondary, not just primary, structure of expression platforms will provide critical insight to guide experimental work. While amending annotations to include features of expression platform would be arduous, it would add great momentum to understanding riboswitch function and the development of riboswitch-based applications for research and medicine.

## Conclusion and perspectives

Despite not being all-encompassing, we believe that this mechanistic framework is useful for conceptualizing the common principals coupling aptamer occupancy and regulatory responses. However, challenges remain to fully unify this integrative perspective. Extensive effort has been dedicated to understanding the structures and ligand-binding properties of aptamer domains. Indeed, these RNAs serve as model systems for many RNA-centered research efforts, including the development of robust computational methods for tertiary RNA folding, establishing new approaches for targeting RNA with small molecule therapeutics, and as modules for new RNA devices (22). While there is further progress to be made in understanding the coupling of ligand binding and gene regulation, we hope that the reframing of riboswitch classification presented in this review will provide the concepts and terminology to discuss, investigate, and deepen our collective understanding of riboswitch biology. An important advance to this knowledge will be the application of bioinformatic approaches to comprehensively annotate riboswitch expression platforms, including information such as sequence conservation, and secondary structure including and beyond the structural switch and RNAP pause sites. However, it is likely that some of this important sequence information is sparsely used or even idiosyncratic to a single bacterial species (*e.g.*, the previously mentioned “que pause” in the *B. subtilis* (29)). This makes their identification and annotation by standard bioinformatic approaches difficult, requiring the need for new computational methods. One potential path forward may be found in machine learning tools, which have been used for species determination (177), prediction of evolutionary traits (178), gene prediction (179), splice-site identification (180), and tuning the performance of synthetic riboswitches (181).

Given that riboswitches are becoming increasingly appreciated as therapeutic targets (182, 183, 184, 185, 186), a mechanism-based perspective of riboswitches, inclusive with respect to genetic regulation, will widen the scope for design and discovery of RNA-targeting small molecules. Further, recent advances in the use of riboswitches to control gene expression in human cells (187, 188) have opened new therapeutic avenues for regulation using small molecule-responsive RNAs. However, effective and safe implementation requires a much deeper understanding of the mechanisms by which the RNA converts small molecule binding to biological outcomes. For these efforts to advance, ongoing work towards developing a detailed mechanistic understanding of the relationship between the aptamer domain and the expression platform of natural and synthetic riboswitches is required.

## Conflict of interest

R. T. B. serves on the Scientific Advisory Boards of Expansion Therapeutics, SomaLogic and MeiraGTx. All other authors declare that they have no conflicts of interest with the contents of this article.
